# Gum Arabic nanoformulation rescues neuronal lesions in bromobenzene-challenged rats by its antioxidant, anti-apoptotic and cytoprotective potentials

**DOI:** 10.1038/s41598-022-24556-0

**Published:** 2022-12-08

**Authors:** Hailah M. Almohaimeed, Hanan Waly, Nasser S. Abou Khalil, Khaled M. A. Hassanein, Basal Sulaiman M. Alkhudhairy, Elham A. Abd-Allah

**Affiliations:** 1 Department of Basic Science, College of Medicine, Princess Nourah bint Abdulrahman University, Riyadh 11671, P.O. Box 84428, Saudi Arabia; 2 Laboratory of Physiology, Department of Zoology, Faculty of Sciences, Assiut University, Assiut, Egypt; 3 Department of Medical Physiology, Faculty of Medicine, Assiut University, Assiut, 71526 Egypt; 4 Department of Pathology and Clinical Pathology, Faculty of Veterinary Medicine, Assiut University, Assiut, Egypt; 5 College of Medicine, Shaqra University, Riyadh 7396, P.O. Box 13343, Saudi Arabia; 6 Department of Zoology, Faculty of Science, New Valley University, EL-Kharga, Egypt

**Keywords:** Biological techniques, Neuroscience

## Abstract

Bromobenzene (BB) is a hazardous environmental contaminant because of its multiple routes of exposure and the toxicity of its bio-derivates. It could elicit neuronal alterations by stimulating redox imbalance and apoptotic pathways. Gum Arabic (GA) protected the hippocampus of a type 2 diabetic rat model from cognitive decline. Whether gum Arabic nanoemulsion (GANE) can increase the neuroprotectant potency of GA in fighting BB-associated neurological lesions is the question to be answered. To accomplish this objective, 25 adult male Wistar rats were randomly and equally assigned into five groups. Control received olive oil (vehicle of BB). BB group received BB at a dose of 460 mg/kg BW. Blank nanoemulsion (BNE) group supplemented with BNE at 2 mL of 10% w/v aqueous suspension/kg BW. GANE group received GANE at a dose of 2 mL of 10% w/v aqueous suspension/kg BW. BB + GANE group exposed to BB in concomitant with GANE at the same previous doses. All interventions were carried out daily by oral gavage for ten consecutive days. BB caused a marked increase in malondialdehyde and succinate dehydrogenase together with a marked decrease in reduced glutathione, glutathione peroxidase, glutathione reductase, superoxide dismutase, catalase, and lactate dehydrogenase in the brain. BB was accompanied by pathological deteriorations, amyloidosis, and reduced immuno-expression of integrase interactor 1 in the hippocampal region. Administration of GANE was beneficial in reversing the aforementioned abnormalities. These results pave the road for further discovery of nano-formulated natural products to counter the threats of BB.

## Introduction

Bromobenzene (BB), an industrial solvent used in the production of the synthetic intermediate phenyl magnesium bromide, in addition to its use as an additive in motor oils and as a flame retardant^1^. It is frequently present in table-ready foods as contaminants residues and in high-fat foods, e.g. sandwich cookies and cake doughnuts^2,3^. The majority of experimental animal studies about BB focus on its adverse effects on hepatic and renal microenvironment that are mediated by initiation of lipid peroxidation, inhibition of enzymatic antioxidants, and shifting the cell fate decisions to the anti-survival events^4–6^.

The brain is highly vulnerable to peroxidative damage as it has a relative abundance of redox-active transition metals, enrichment of unsaturated lipid, dependence on calcium signaling, autoxidation of neurotransmitters, and low stock of antioxidant enzymes^7–9^. Although these biological features make the brain one of the top-ranked target organs for BB burden, there is no data in the literature up to our knowledge about this issue.

The application of natural antioxidant cytoprotective agents having a broad safety window is of utmost significance in fighting the adverse effects of BB on xenobiotics-associated neural disturbances. Gum Arabic (GA) is a dried exudate from trunks and branches of acacia trees, especially* Acacia senegal* and* Acacia seyal*, family Fabaceae^10^. It composes of galactose backbone joined by side chains of arabinose, galactose, rhamnose and (methyl-) glucuronic acid^11^. It has a pronounced economic impact on the pharmaceutical and food industry as an emulsifier and a stabilizer^12^. From the biological viewpoint, GA elicits contradictory neuroprotective outcomes. Although it exhibited a promising role in hindering learning and memory loss in a type 2 diabetic rat model, it failed to counteract the oxidative stress-induced deletion of hippocampal pyramidal neurons in electromagnetic irradiated rats^13,14^. Besides the difference in the dose/duration experimental schedule, the failure of GA to provide a full neuroprotectant potency might partially emerge from low bioavailability, short half-life, and non-specific targeting^15^. Nanomedicine has the potential to offer solutions to avoid these obstacles so much so that a new wave of researches called “green chemistry” has been emerged strongly on the surface of literature encouraging using the natural product-loaded nanocomposites as a hopeful domain to carry the therapeutic cargo. This is driven by the facts that the nano-carriers possess a variety of positive aspects including enhanced solubility, absorption, bioavailability, and controlled-release of drugs^16,17^. Therefore, we hypothesized that the administration of gum Arabic-nanoparticles (GA-NPs) will get benefit from both the active phytochemical constituents of GA and the stability and accessibility of nano-composite to be an effective modulator in blocking the neural lesions induced by BB. To our knowledge, there is no available data regarding the protective effects of GA-NPs against BB-induced neural impairment. Thus, this study aimed to highlight this subject and its underlying mechanisms using an experimental rat model.

## Materials and methods

### Chemicals

Gum Arabic was purchased from a local market in Jeddah, Saudi Arabia, and used in preparation of aqueous extract^18^. The dried gum crushed into a fine powder and stored in airtight plastic containers at 5 °C till used. Gum extract (10% w/v) prepared by soaking 10 g of gum in 100 ml of distilled water for 24 h, then filtering it. It was freshly prepared daily. BB was purchased from Sigma Chemical Co. (St. Louis, MO, USA).

### Preparation of magnetic nanoparticles (MNP)

Ferrous chloride and ferric chloride 10 mM were added to 1.5 M of sodium hydroxide solution under stirring at temperature 75 °C. The mixture was stirring with nitrogen gas protection for one hour. MNP was separated by using magnetic separator and then washed by distilled water for five times^18^.

### Preparation of Gum Arabic magnetic nanoparticles (GA-MNP)

One ml of previously prepared MNP with concentration 10 mg/ml was added to 5 ml of 10% Gum Arabic, the mixture was vortexed for 1 min followed by sonicated for 10 min. The GA-MNP was separated by magnetic separator and washed five times with distilled water^18^.

### Characterization of the Gum Arabic

#### Entrapment efficiency measurement

Dialysis tubing technique was used to purify the synthesized nano-formulations GA for eliminating impurities and free non-conjugated compounds suspended in the solution by eluting it through regenerated cellulose (Amicon 10,000 MWCO ultra filter, Millipore, USA). The Entrapment efficiency (EE%) for GA was measured and processed with the microplate reader (BMG Labtech, Germany). The compound entrapment efficiency was calculated from the ratio of the compound amount incorporated into the NPs to total added compound amount.

### Transmission electron microscopy (TEM)

Particle morphology of GA was examined by transmission electron microscopy (TEM, Philips CM-10, FEI Inc., Hillsboro, OR, USA). 100 μg/mL of the nano-suspension were dropped into formvar-coated copper grids, and after complete drying, the samples were stained using 2% w/v uranyl acetate (Electron Microscopy Services, Ft. Washington, PA). Image capture and analysis were done using Digital Micrograph and Soft Imaging Viewer Software.

### Zeta potential analyses

Zeta potential of NPs were determined by photon correlation spectroscopy (PCS) using a Zeta Sizer (Nano ZS, Malvern Instruments, UK), All the samples were maintained at a constant temperature of 25.0 °C.

### Animals and experimental groups

A total of 25 adult male Wistar rats were used in this study. They were kept in cages situated in a well-ventilated room and maintained under standard laboratory conditions. Food and water were provided ad libitum. After an acclimatization period of one week, rats were randomly and equally divided into five groups as the following:

The control group received olive oil (a vehicle of bromobenzene) at a dose of 1 mL/kg BW.

The bromobenzene (BB) group received BB at a dose of 460 mg/kg BW dissolved in 1 mL olive oil^19^.

The blank nanoemulsion (BNE) group received BNE at a dose of 2 mL of 10% w/v aqueous suspension/kg BW.

The gum Arabic loaded nanoemulsion (GANE) group received GANE at a dose of 2 mL of 10% w/v aqueous suspension/kg BW^20^.

The BB + GANE group received BB in concomitant with GANE at the same previous doses.

All interventions were carried out daily by oral gavage for ten consecutive days. The proposal was reviewed by Institutional Review Board in the Faculty of Medicine, Assuit University, Assuit, Egypt (approval number: IRB17300789). All methods were performed in accordance with the relevant guidelines and regulations. The study is reported in accordance with ARRIVE guidelines.

### Collection and preparation of sample

At the end of the experimental schedule, rats were sacrificed by cervical dislocation under anesthesia using sodium thiopental. One part of the brain was quickly removed and homogenized in 1 ml of 0.1 M phosphate buffer (pH 7.4). The homogenates were centrifuged at 10,000 rpm for 15 min, and the supernatants were kept frozen at − 20 °C for the subsequent biochemical assays. The other part was fixed in 10% neutral buffered formalin for histopathological and immunohistochemical examination.

### Biochemical measurements

Malondialdehyde (MDA) was determined by estimation of thiobarbituric acid reactive substances colorimetrically according to the method of Uchiyama and Mihara^21^. Reduced glutathione (GSH) were estimated by a commercially available kit obtained from Bio-diagnostic Company, Giza, Egypt (catalog number: GR2511). Lactate dehydrogenase (LDH) activity was analyzed by a colorimetric kit purchased from Elabscience Company, USA (catalog number: E-BC-K046-M). Using coenzyme I as a hydrogen carrier, LDH catalyze lactic acid to produce pyruvate. Pyruvate reacts with 2, 4-dinitrophenylhydrazine to form pyruvate dinitrophenylhydrazone, which is red-brown in alkaline solution, and the color depth is proportional to pyruvate concentration and is related to the LDH activity. Succinate dehydrogenase (SDH) activity was evaluated using a colorimetric kit (catalog number: ab228560, Abcam Company, UK). In this method, SDH converts succinate to fumarate, and transfers the electron to an artificial electron acceptor, which changes the color from blue to a colorless product depending upon the enzymatic activity. Superoxide dismutase (SOD) activity was assessed according to its ability to inhibit the autoxidation of epinephrine at alkaline medium following Misra and Fridovich^22^. The catalase (CAT) activity was estimated according to a previous procedure^23^. Briefly, CAT reacts with a known quantity of hydrogen peroxide. The reaction is stopped after exactly one minute with catalase inhibitor. In the presence of peroxidase, the remaining hydrogen peroxide reacts with 3,5-Dichloro-2-hydroxybenzene sulfonic acid and 4-aminophenazone to form a chromophore whose color intensity is inversely proportional to the amount of CAT in the sample. Glutathione peroxidase (GPx) and glutathione reductase (GR) were measured by colorimetric kits obtained from Abcam Company, UK (catalog number: ab102530 and ab83461, respectively). Protein concentration was determined according to the method described by Lowry et al*.*^24^. All measured parameters in the brain homogenate were corrected with the corresponding total protein concentrations. All biochemical estimations were carried out using Unicam 8625 UV/V spectrophotometer (Cambridge, UK).

### Histopathological examination

Tissue samples from the brain (hippocampus region) were fixed in 10% neutral buffered formalin, dehydrated by ascending grades of alcohol, cleared by xylene, and embedded in paraffin. The tissues were sectioned (5 microns in thickness) and stained with hematoxylin and eosin (H&E)^25^. Congo Red Stain was used as a marker for amyloid deposition in brain tissues. It was fixed in bouin’s solution and processed, dehydrated, embedded, sectioned, and stained according to standard procedures with some modifications^26^.

### Immunohistochemistry of integrase interactor 1 (INI-1)

Paraffin sections of the brain were cleared in xylene, rehydrated in graded ethanol, immersed in water for 5–10 min, and incubated in 0.3% H_2_O_2_ in 70% methanol for 20 min to inhibit endogenous peroxidase activity. Then, specimens were rinsed three times for 5 min in phosphate-buffered saline solution (PBS), and epitopes were unmasked by boiling in citrate buffer (pH 6.0) for 10–15 min. After rinsing in PBS, the sections were blocked for 30–60 min in 3% bovine serum albumin (BSA) in PBS, and incubated with primary antibody against INI-1 in 0.1% BSA in PBS overnight at 4 °C in a humidified chamber. Brain samples were then rinsed in PBS and incubated with 7.5 g/mL of biotinylated secondary antibody in 0.1% BSA in PBS for 1 h at room temperature, followed by avidin–biotin amplification (ABC Elite) for 30 min, and were developed with 3,3-diaminobenzidine peroxidase substrate. Sections were counterstained with Mayer hematoxylin for 3 min and mounted. Negative controls were obtained by substituting the primary antibody with PBS^27^.

### Detection of apoptosis by staining with acridine orange

The procedure was performed according to that of Sayed and et al*.*^28^. To prepare a stock solution of acridine orange (AO), 50 mg acridine orange was dissolved in 10 ml of distilled water and stored in the refrigerator (0.5% AO). One ml of AO stock solution and 0.5 ml of glacial acetic acid were added to 50 ml of distilled water. The pH of the staining solution was approximately 3 and the AO concentration was 0.01%. Then, 5 μm paraffin sections were dewaxed (two times for 30 min) and rehydrated in a descending series of ethanol (100, 95, and 70%) and distilled water. Dried sections of glass slides were put in a trough with AO staining solution (0.01%). After two minutes of staining, the slides are washed gently with distilled water, dried and then analyzed using a Leitz DM 2500 microscope with the external fluorescent unit Leica EL 6000.

### Statistical analysis

Data were represented as mean ± standard error of the mean (SEM). The results were analyzed by one-way analysis of variance (ANOVA) followed by Duncan post-test using SPSS program version 16 (SPSS Inc., Chicago, USA). Differences of *P* < 0.05 were considered to be statistically significant.

## Results

### Characterization of modified GA with magnetite and chitosan nanoparticles

#### Magnetic GA nanocomposites

**Figure 1:**
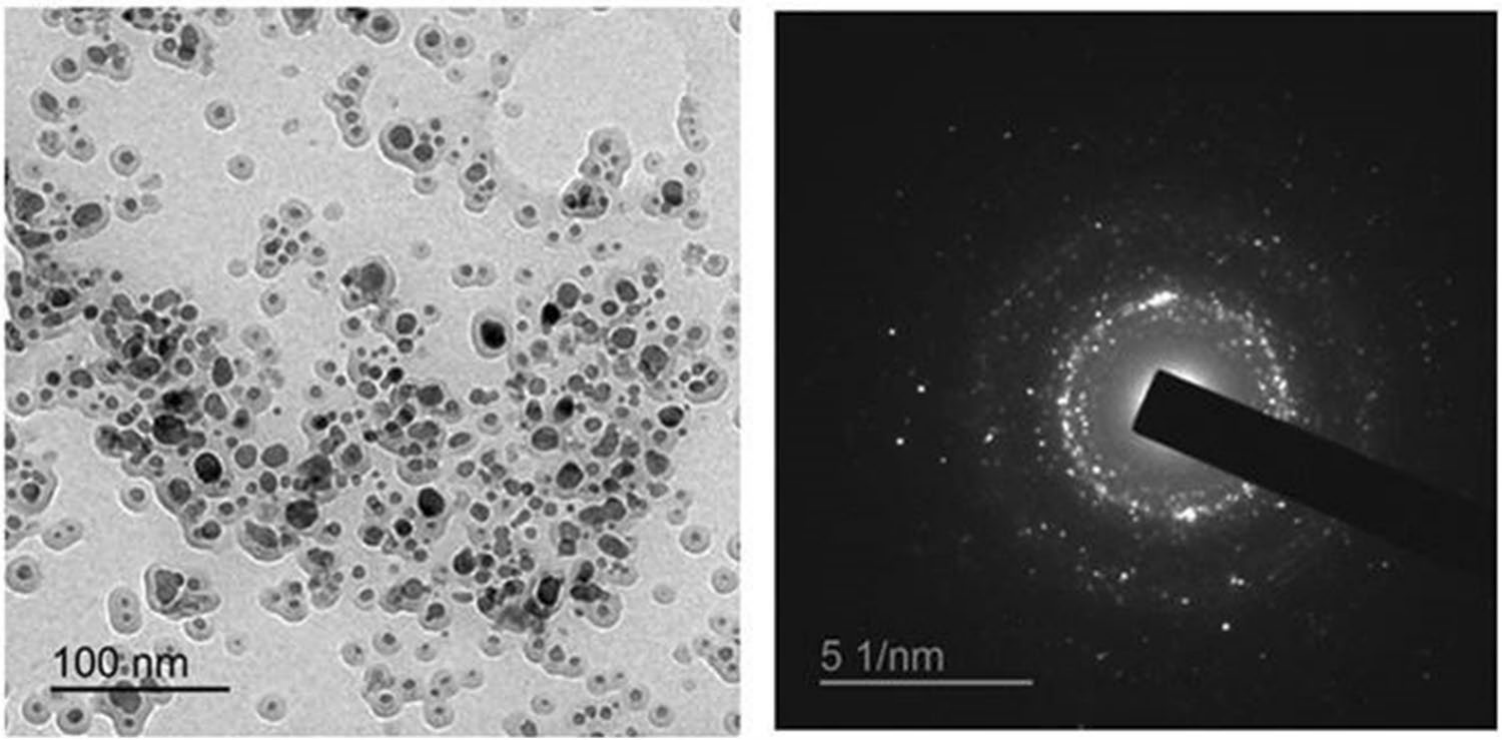
Examination of Magnetic GA nanocomposites by HR-TEM.

High Resolution Transmission Electron Microscopy (HR-TEM) displayed that the average size of magnetic GA nanocomposites was ranged from 9.17 to 13.22 nm (Fig. 1). The selected area electron diffraction (SAED) pattern demonstrated a highly crystalline structure of magnetic GA nanocomposites.

#### Fourier-transform infrared spectroscopy (FTIR)

**Figure 2:**
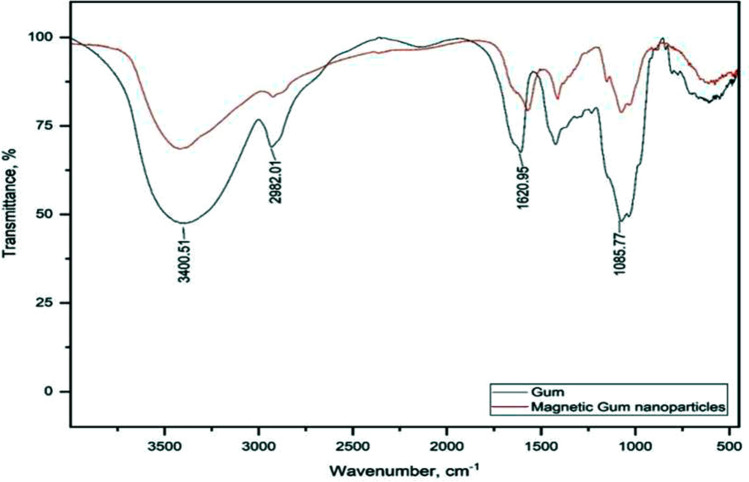
FTIR spectra for GA and magnetic GA linked with chitosan nanoparticles.

FTIR spectra for GA and magnetic GA linked with chitosan nanoparticles were shown in Fig. 2. The peak corresponds to C–H vibration at 2982 cm^−1^ was decreased after linked with magnetite and chitosan nanoparticles. Also, peak at 1620 cm^−1^ which corresponds to carboxylate group was decreased due to interaction of –NH2 group with –COO– group. Strong peak appeared at 1085 cm^−1^ which related to C–O stretching was decreased after modification with magnetite and chitosan nanoparticles.

#### Zeta Potential

Zeta potential analysis of GA functionalized with magnetite coated with chitosan nanoparticles was + 48.5 mV.

### The biochemical findings in the brain

As shown in Table 1, the brain of BB intoxicated rats was characterized by a significant increase in MDA concomitant with a significant decrease in GSH, GPx, GR, SOD, and CAT compared to the control group. GSH, SOD, and SDH in BNE were significantly higher than in the control group. However, the LDH in the BNE group was significantly lower than in the control group. The comparison between MDA, GPx, GR, and CAT of the BNE group and those of the control group revealed an absence of significant difference. A significant increase in GPx, GR, SOD, CAT, LDH, and SDH was observed when comparing the GANE and control groups. On the contrary, no significant difference could be found when comparing the MDA and GSH of the GANE group and those of the control group. GANE dosing in BB neurotoxic rat model resulted in a significant reduction in the MDA and LDH and a significant elevation in the GSH, GPx, GR, SOD, CAT, and SDH compared to the BB group. However, LDH and MDA in the BB + GANE group were still significantly higher than in the control group. The GANE intervention is highly effective to a degree that GSH, GPx, GR, and CAT were significantly higher than those in the control group. While SOD and SDH of the BB + GA group were not significantly changed relative to those of the control group.

**Table 1:** Effects of gum Arabic nanoformulation on redox potential, and marker enzymes of mitochondrial respiration and glycolysis in the brain of adult male rats challenged by bromobenzene.

Group	Control	BB	BNE	GANE	BB + GANE
*Parameter*					
MDA level (Mol/ mg protein)	2.300 ± 0.082^c^	6.625 ± 0.769^a^	1.825 ± 0.132^c^	2.475 ± 0.111^c^	3.675 ± 0.132^b^
GSH level (mmol/mg protein	79.405 ± 1.894^c^	37.813 ± 1.655^d^	91.288 ± 3.531^b^	80.470 ± 2.893^c^	111.010 ± 3.548^a^
GPx activity (µmol/min/mg protein	243.360 ± 3.197^c^	159.220 ± 4.838^d^	253.460 ± 7.906^bc^	259.400 ± 3.460^b^	327.240 ± 2.006^a^
GR activity (µmol/mg protein)	11.110 ± 0.577^c^	6.593 ± 0.199^d^	11.238 ± 0.553^c^	14.423 ± 1.075^b^	16.940 ± 0.651^a^
SOD activity (U/min/mg protein)	0.528 ± 0.007^c^	0.227 ± 0.012^d^	0.567 ± 0.010^b^	0.626 ± 0.007^a^	0.553 ± 0.012^bc^
CAT activity (U/min/mg protein)	1.606 ± 0.021^c^	0.854 ± 0.011^d^	1.678 ± 0.011^bc^	1.735 ± 0.010^b^	1.860 ± 0.048^a^
LDH activity (nmol/min/mg protein)	441.110 ± 7.440^d^	828.980 ± 5.980^a^	421.590 ± 4.052^e^	507.470 ± 4.815^c^	533.960 ± 4.684^b^
SDH activity (mmol/mg protein)	15.293 ± 0.901^b^	8.345 ± 0.461^c^	19.595 ± 0.755^a^	20.605 ± 2.034^a^	17.815 ± 0.694^ab^

*GA* gum Arabic; *BB* bromobenzene; *BNE* blank nanoemulsion: *GANE* gum Arabic loaded ananoemulsion; *BB* + *GANE* bromobenzene + gum Arabic loaded nanoemulsion.

*MDA* malondialdehyde; *GSH* reduced glutathione; *GPx* glutathione peroxidase; *GR* glutathione reductase; *SOD* superoxide dismutase; *CAT* catalase; *LDH* lactate dehydrogenase; *SDH* succinate dehydrogenase.

Results are expressed as the mean ± SEM of 5 rats per group.

^a–d^Different letters indicate significant difference at *p* < 0.05 (one-way ANOVA followed by Duncan post-test).

### The histopathological findings

**Figure 3:**
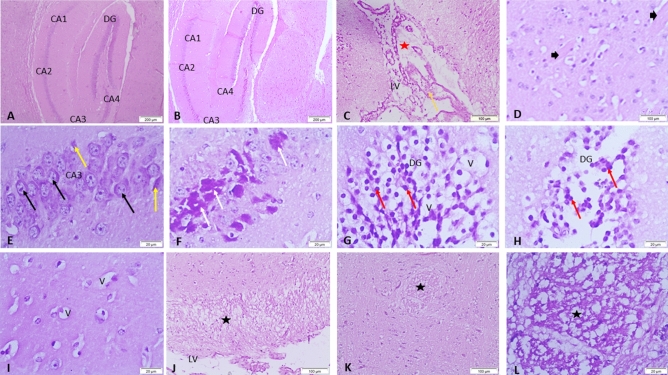
Representative micrograph of the control olive oil group showing the hippocampal region in the form of CA1, CA2, CA3 & CA4 regions and dentate gyrus (DG) is seen surrounding CA4 (**A**). (**B**–**L**); Representative micrograph of the bromobenzene group showing pathological changes in the hippocampal region, edema (red star) and congestion in blood vessels (orange arrow) in lateral ventricle (LV), amyloid substance (arrow heads), in CA3 region shows neurons with prominent nuclei (black arrows), apoptosis (yellow arrows), neuronal necrosis (white arrows), in DG region shows vacuolation (V) and apoptosis (red arrows), areas of extensive myelin vacuolation adjacent to LV with vacuolar clefts are typically empty and represent myelin sheath splitting (black stars). HE.

**Figure 4:**
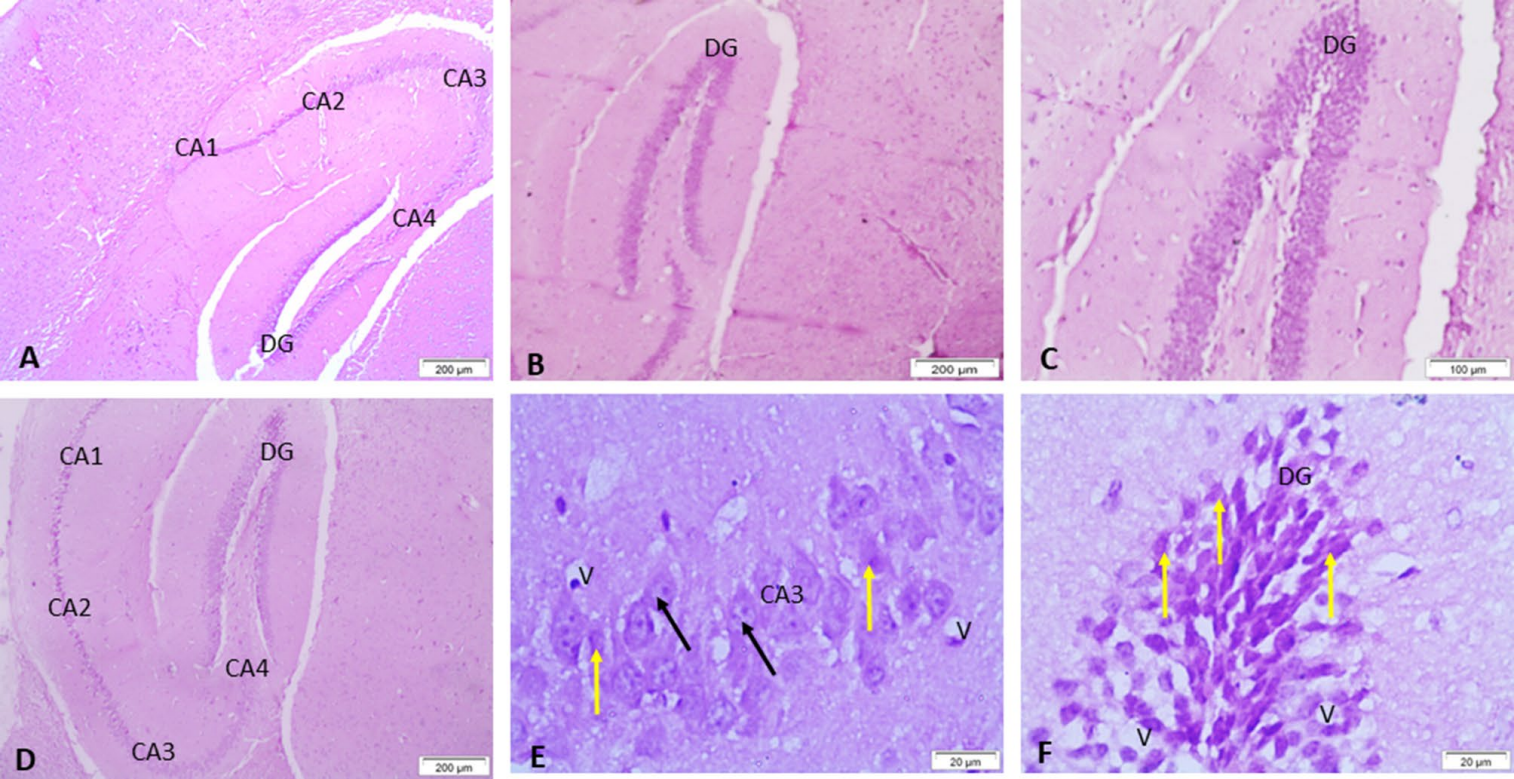
Representative micrograph of the blank nanoemulsion group showing normal the hippocampal region in the form of CA1, CA2, CA3 & CA4 regions and dentate gyrus (DG) (**A**); Normal hippocampal region in gum Arabic loaded nanoemulsion group (**B**, **C**). (**B**–**L**); bromobenzene + gum Arabic loaded nanoemulsion group showing some pathological changes in the hippocampal region in CA3 region including neurons with prominent nuclei (black arrows), apoptosis (yellow arrows), neuronal necrosis (white arrows), DG region shows vacuolation (V) and apoptosis (yellow arrows) (**D**–**F**). HE.

**Figure 5:**
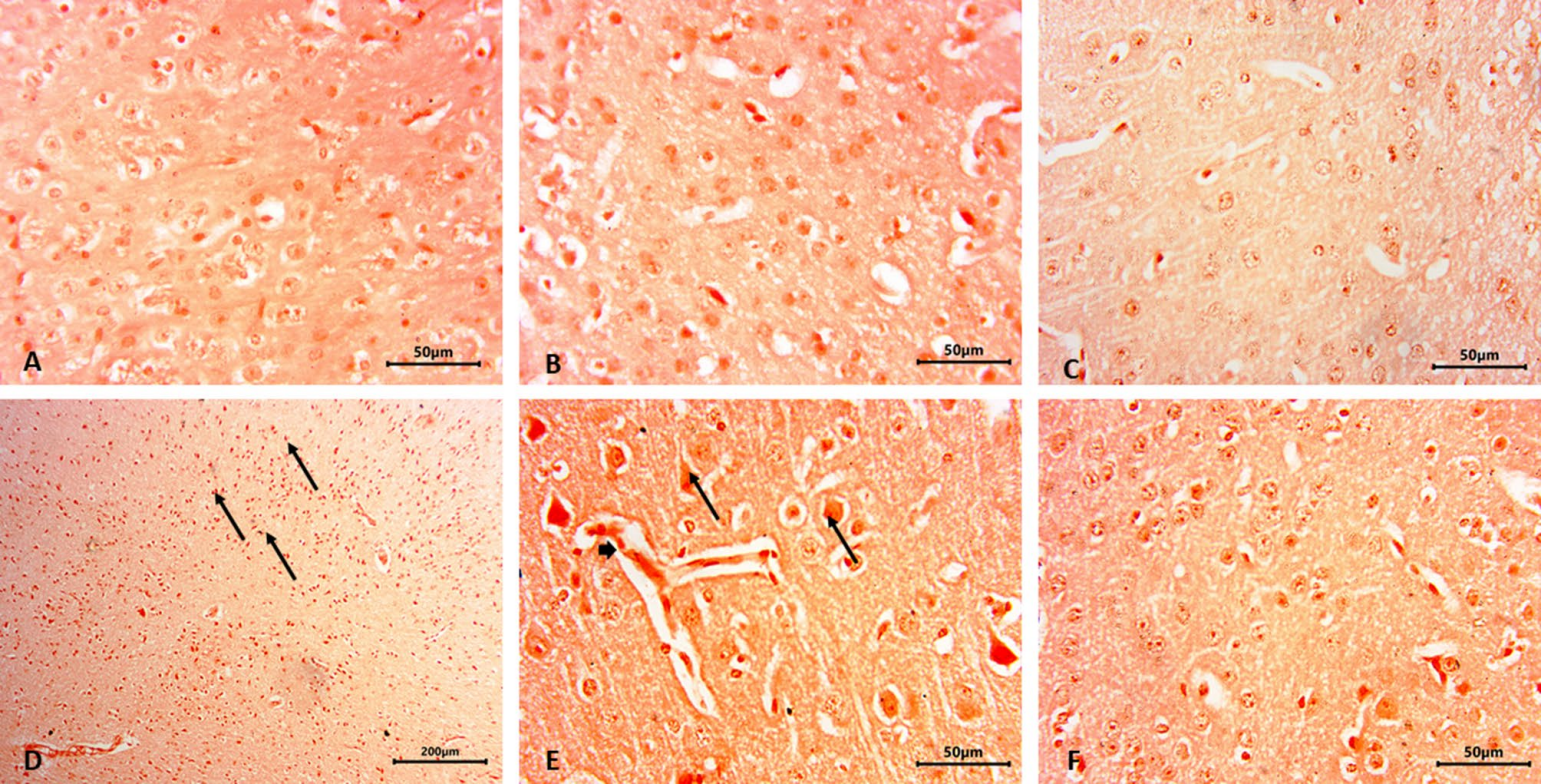
Representative micrograph of the control olive oil group, blank nanoemulsion, and gum Arabic loaded nanoemulsion groups, respectively with no amyloid precipitate with congo red (**A**–**C**). Amyloid staining labels the vessels (arrow head), the amyloid plaque (arrows) presents in bromobenzene group (**D**, **E**), mild deposits in bromobenzene + gum Arabic loaded nanoemulsion group (**F**). Congo-red stain.

The lesion scores of the histopathological outcomes of the brain hippocampal region in all studied groups were illustrated in (Table 2). Histopathological examination of the control (Fig. 3A), BNE (Fig. 4A), GANE (Fig. 4B,C) groups revealed the normal appearance of the hippocampal region in the form of (Cornu Ammonis) CA1, CA2, CA3, CA4 regions and dentate gyrus (DG) was observed surrounding CA4. While the BB group showed pathological changes in the hippocampal region as edema and congestion in blood vessels of the lateral ventricle (LV). Amyloid plaques were demonstrated in the blood vessels of hippocampal region. CA3 region showed pyramidal neurons with prominent nuclei, swollen neurons, neuronal vacuolation, apoptosis, and many areas revealed neuronal necrosis. Also, neuronal vacuolation, apoptosis, and neuronal necrosis were seen in the DG region. Multiple focal areas of extensive myelin vacuolation adjacent to LV with vacuolar clefts were typically empty and represented myelin sheath splitting (Fig. 3B–L). The BB + GANE group revealed improvement of the histopathology of the hippocampal region except minor some pathological changes in CA3 region neurons as necrosis, vacuolation and apoptosis in DG region (Fig. 4D–F). Amyloidosis was confirmed by Congo-red stain which revealed negative reaction in the control, BNE, and GANE groups. The BB group revealed strong reaction labels on the vessels wall and amyloid plaque while mild deposits were observed in the BB + GANE group (Fig. 5).

**Table 2:** The lesion scores of the histopathological results of the brain hippocampal region in all experimental groups.

Groups	Control	BB	BNE	GANE	BB + GANE
*Lesions*					
Edema in LV	−	+ +	−	−	+
Congestion of blood vessels in LV	−	+ +	−	−	−
Neuronal vacuolation	−	+ + +	−	−	+
Apoptosis in CA3	−	+ + +	−	−	+ +
Apoptosis in DG	−	+ + +	−	−	+
Necrosis in CA3	−	+ + +	−	−	−
Amyloid precipitation	−	+ +	−	−	+
Myelin vacuolation	−	+ + +	−	−	−

*GA* gum Arabic; *BB* bromobenzene; *BNE* blank nanoemulsion: *GANE* gum Arabic loaded ananoemulsion; *BB* + *GANE* bromobenzene + gum Arabic loaded nanoemulsion; *LV* lateral ventricle; *CA3* Cornu ammonis3; *DG* dentate gyrus.

− No lesions, + lesions present in 2–4 sections, +  + lesions present in 5–7 sections, +  +  + lesions present in 8–12 sections.

### Immuno-expression of integrase interactor 1 (INI-1)

**Figure 6:**
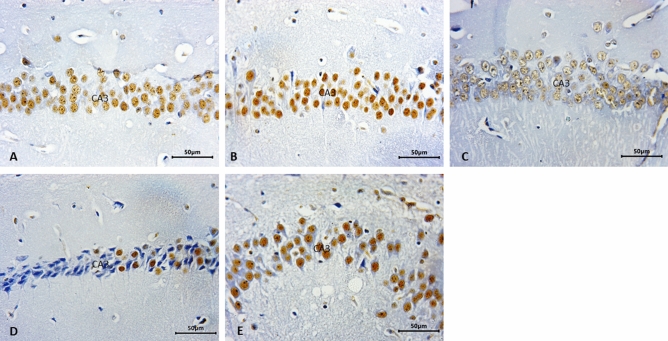
Representative micrograph INI-1 expression in CA3 showing strong expression in the control olive oil group (**A**), blank nanoemulsion group (**B**), gum Arabic loaded nanoemulsion group (**C**) and bromobenzene + gum Arabic loaded nanoemulsion group (**D**). Weak reaction in bromobenzene group (**E**).

INI-1 expression in CA3 revealed strong expression in the control, BNE, GANE, and BB + GANE groups. While weak expression of INI-1 in BB group (Fig. 6).

### Apoptotic detection

**Figure 7:**
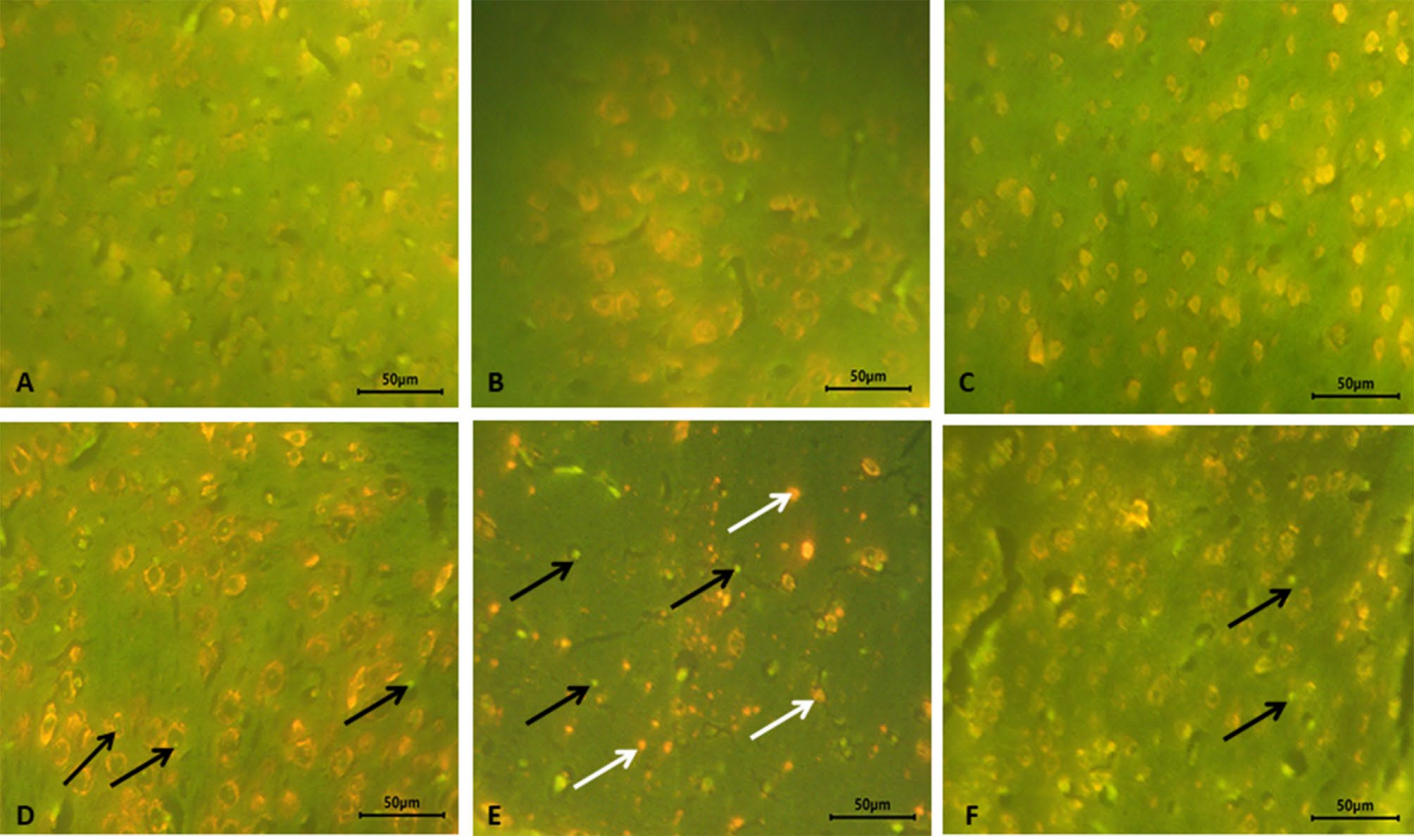
Representative micrograph of the control olive oil, blank nanoemulsion, and gum Arabic loaded nanoemulsion groups, respectively. Cells appear normal with a green nucleus (**A**–**C**). Early apoptotic cells with a bright green nucleus and condensed chromatin (black arrows), and late apoptotic cells with condensed orange chromatin (white arrows) present in bromobenzene group (**D**, **E**), A few early apoptotic cells (black arrows) in bromobenzene + gum Arabic loaded nanoemulsion group (**F**). Acridine orange stain.

We stained the neuronal cells with acridine orange to investigate the extent of programmed cell death (Fig. 7). Cells appeared normal with a green nucleus (Fig. 7A–C) in the control olive oil, BNE, and GANE groups. On the other side, BB group was characterized by the presence of early apoptotic cells with a bright green nucleus and condensed chromatin, and late apoptotic cells with condensed orange chromatin (Fig. 7D–E). GANE succeeded in reducing the apoptosis manifested by appearance of a few early apoptotic cells (Fig. 7F).

## Discussion

The marked decrease in brain SOD and CAT activities is in the same line as that found in the hepatic and renal tissues of the mice model^29^. When GSH is depleted under BB burden, the electron flow in the respiratory chain was blocked in parallel with an increase in emission of free radicals which consumes the enzymatic antioxidants^30^. For instance, surplus formation of hydrogen peroxide inhibits the enzymatic activity of SOD by oxidation of proline and histidine residues proximal to the active site copper atom^31^.

There is a reciprocal relationship between the deposition of amyloid plaques and oxidative stress. Amyloidosis is strongly linked to oxidative stress as redox-active metal ions act as a catalyst when they interact with amyloid proteins stimulating the production of reactive oxygen species. This outcome also extends to the other surrounding macromolecules causing peroxidative damage^32^. On the other hand, the accumulation of end-products of lipid peroxidation promotes modifications of the γ-secretase complex components, predisposing to amyloidosis^33^.

BB metabolites bind to glutathione, impairing the shield against reactive oxidants and the detoxification of xenobiotics^29,34^. This initiates a lipid peroxidation cascade and alters calcium homeostasis leading to cytotoxicity^35,36^. Its metabolites also generate a variety of derivatives causing cell necrosis^37^. Inhibition of brain SOD in our experimental model led to an accumulation of superoxide anion that reacts with nitric oxide to form peroxynitrite inactivating mitochondrial proteins^38,39^. It is well known that ATP depletion causes necrosis^40^. BB increases lysosomal enzyme activities and disrupts lysosomal membrane stability^4^. This scenario has adverse consequences on the cytological architecture of neurons in the form of hydropic degeneration and necrosis.

BB promoted hepatic nitric oxide production which is implicated in congestion and edema as it acts as a vasodilator agent^41^. The hyperlactatemia which resulted from increased LDH activity could be involved in decreased resistance of vascular bed with subsequent engorgement of blood in capillaries and transduction of fluid into the tissue spaces^42^.

Neural apoptosis in the BB group is matched with that observed in hepatic and renal tissues owing to the increased transcript level of Bax and decreased those of Bcl-2 and caspase-3^6,29^. GSH depletion inhibits plasma membrane Ca^2+^-ATPases to increase the intracellular calcium pool which elicits diverse cytotoxic pathways^43,44^. Lactic acidosis secondary to increased LDH activity translocates BNIP3 into the mitochondrial membrane, thereby the mitochondrial permeability transition pore opens. Consequently, mitochondria discharge the pro-apoptotic factors such as cytochrome c^45^.

The inhibition in the brain glutathione redox system in the BB group is following a previous scholarly article focusing on BB-induced hepatotoxicity^41^. Cytochrome-mediated BB bio-transformation emits bromobenzene-3, 4-oxide which is sequestered by binding with GSH blocking mitochondrial respiratory function^46^. This fact is reinforced by the inhibition of brain SDH, an enzyme responsible for energy production via its participation in oxidative phosphorylation and the tricarboxylic acid cycle^47^. A previous study in Wistar albino rats reported a marked decrease in the activity of hepatic mitochondrial enzymes, including SDH, following BB toxicity. The inhibition of the mitochondrial respiratory chain worsens the condition by triggering a vicious cycle of production of reactive oxidants^29^. The presence of amyloid plaques could be implicated in the inhibition of mitochondrial key respiratory enzymes^48^. The drop in the level of GPx in the brain of BB-challenged rats may be attributed to its consumption in reducing lipid hydroperoxide to stable non-radical lipid alcohols^41^.

MDA significantly increased in the brain tissue of BB-challenged rats as observed earlier in the hepatic and renal tissues secondary to over generation of free radicals by attacking polyunsaturated fatty acids^49–51^. This outcome has adverse sequelae on genomic stability by creating MDA-induced DNA interstrand cross-links, and on protein functionality by inducing several oxidative modifications^52,53^. Accumulation of MDA reduced cortical neuronal viability and induced cellular apoptosis as well as necrosis^54^.

Increased activity of LDH in brain tissue following BB burden indicates mitochondrial dysfunction, a situation that requires acceleration of glycolysis to keep cells from dying in case of energy shortage^55^. Excessive generation of reactive oxidants induces the expression of glycolytic genes and suppresses the tricarboxylic acid cycle^56^. The re-oxidation of cytosolic NADH by delivering reducing equivalents from this molecule into the electron transport chain sustains glycolysis. The activation of the glycolytic pathway in concomitant with the inhibition of the tricarboxylic acid cycle is one of the key metabolic features of Alzheimer’s disease^56,57^. The disturbance in glucose metabolism is critical in the pathogenesis of Alzheimer’s disease as it can lead to hyperphosphorylation of tau (a microtubule‐associated protein), and induce amyloidogenic derivatives^58^. A mutual connection exists between oxidative stress and LDH activity. The increase in LDH in the BB group suggests an increase in lactate production which motivates reactive oxygen species burst^59^. On the other side, the increased extracellular activity of LDH, which is an intracellular enzyme, is the result of disrupted cell membrane integrity which occurs secondary to free radicals-induced lipid peroxidation^60^. In addition, oxidative stress motivated glycolysis to compensate for the energy supply^61^.

Integrase interactor 1 (INI1) gene encodes a member of the switch/sucrose nonfermenting chromatin remodeling complex, which negatively regulates the cell cycle, modulates cytoskeleton organization, and functions as a tumor suppressor effector^62^. Homozygous deletion of both alleles of the INI1 gene or deletion of one allele and mutation of the second allele results in the development of tumors of the central nervous system^63^. Although there is no histological evidence of carcinogenesis, the lack of immunoreactivity with monoclonal antibodies against the INI1 protein in the BB group, according to our findings, suggested an increase in susceptibility or an early sign of soft tissue sarcoma. However, further examination of oncogenic factors is warranted before reaching this radical conclusion. Oxidative stress following BB exposure could be one of the causative factors contributing to cancer progression by amplifying genomic instability^64^. From another perspective, SDH deficiency in the BB group results in the accumulation of succinate which activates the transcription of target oncogenes related to cell proliferation^65^. High LDH content in the neural cells adds additional dimension as it is directly associated with up-regulated hypoxia-inducible factors pathway that is responsible for extramural invasion, nodal and distant metastases and linked to aggressive phenotypes of cancer^66^.

Although the literature deals with GA as a coating material in nanoparticle delivery system to improve the stability and solubility of bioactive phytochemicals, its nanoparticle formulation is supposed to enhance permeability and retention effect, and precise targeting of GA and improve its antioxidant and cytoprotective efficacy^67^. The use of GA as a nanocarrier for nutritional products complicates the isolation of individual biological effects of GANP away from portable materials. The increase in antioxidant activities in the brain homogenate following GANP treatment is corresponding to the antioxidant ability of GA in fighting redox disturbance in the testis and liver of alloxan diabetic rats^68,69^. This finding could be due to an increase in their transcript levels by post-translational modification. The reduction in the brain content of MDA is similar to that observed in the ovary when mice high fat diet feeding mice administrated GA^70^. This is most probably attributed to the free radical scavenging properties of GA^71^.

GA succeeded in shifting metabolic pathways in the brain from anaerobic glycolysis to aerobic respiration. The increase in LDH activity in the BB + GA group is matched with a rat model of doxorubicin-induced cardiotoxicity^72^. In contrast, the activity of testicular LDH and transcript abundance of glucose transporters increased following the supplementation of diabetic rats with GA^73^. The difference in the experimental model might underlie this contradiction.

Strong immuno-expression of INI1 in the hippocampus of the BB + GANP group reflects the anti-cancer nature of GA owing to its genoprotective and antioxidant properties. GA is a potent inhibitor of cell transformation, oncogenesis, and metastasis by decreasing ß-catenin and vav-3-oncogene expression^74,75^. The amelioration in neuronal apoptosis following GANP intervention is in the same line with previous research that revealed that GA is effective in down-regulation the gene expression of caspase-3 and in protecting against DNA damage in the testicular tissue of cisplatin intoxicated rats^76^.

## Conclusion

BB-induced oxidative stress and metabolic disturbances in the brain of rats concurrent with histopathological deteriorations, amyloid plaque deposition, and INI1 down-regulation in the hippocampal region. These changes led to hippocampal atrophy and cognitive impairment, and in the long-term may predispose to dementia and Alzheimer's disease. Supplementation of BB-challenged rats with GANE was beneficial in reversing the above-mentioned abnormalities. These findings provide promising prospects guiding future investigations and the discovery of nano-formulated natural products to counteract the hazards of BB. The main limitations in the current study are the lack of behavioral tests to explore the cognitive impairments, such as Y-maze spontaneous alternation and object recognition tests.

## Data Availability

The datasets analyzed during the current study available from the corresponding author on reasonable request.
